# Model diagnostics and refinement for phylodynamic models

**DOI:** 10.1371/journal.pcbi.1006955

**Published:** 2019-04-05

**Authors:** Max S. Y. Lau, Bryan T. Grenfell, Colin J. Worby, Gavin J. Gibson

**Affiliations:** 1 Department of Ecology and Evolutionary Biology, Princeton University, New Jersey, USA; 2 Fogarty International Center, National Institute of Health, Bethesda, MD, USA; 3 Broad Institute, Cambridge, MA 02142, USA; 4 Maxwell Institute for Mathematical Sciences, School of Mathematical and Computer Sciences, Heriot-Watt University, Edinburgh, EH14 4AS, UK; Duke University, UNITED STATES

## Abstract

Phylodynamic modelling, which studies the joint dynamics of epidemiological and evolutionary processes, has made significant progress in recent years due to increasingly available genomic data and advances in statistical modelling. These advances have greatly improved our understanding of transmission dynamics of many important pathogens. Nevertheless, there remains a lack of effective, targetted diagnostic tools for systematically detecting model mis-specification. Development of such tools is essential for model criticism, refinement, and calibration. The idea of utilising *latent residuals* for model assessment has already been exploited in general spatio-temporal epidemiological settings. Specifically, by proposing appropriately designed non-centered, re-parameterizations of a given epidemiological process, one can construct latent residuals with known sampling distributions which can be used to quantify evidence of model mis-specification. In this paper, we extend this idea to formulate a novel model-diagnostic framework for phylodynamic models. Using simulated examples, we show that our framework may effectively detect a particular form of mis-specification in a phylodynamic model, particularly in the event of superspreading. We also exemplify our approach by applying the framework to a dataset describing a local foot-and-mouth (FMD) outbreak in the UK, eliciting strong evidence against the assumption of no within-host-diversity in the outbreak. We further demonstrate that our framework can facilitate model calibration in real-life scenarios, by proposing a within-host-diversity model which appears to offer a better fit to data than one that assumes no within-host-diversity of FMD virus.

## Author summary

Integrated modelling of conventional epidemiological data and modern genomic data (i.e. phylodynamics) has made significant progress in recent years, due to the ever-increasing availability of genomic data and development of statistical methods. However, there is a lack of tools for carrying out effective diagnostics for phylodynamic models. We propose a novel model diagnostic framework that involves a latent residual process which is *a priori* independent of model assumptions and which can be used to quantify, and reveal the nature of, model inadequacy. Our results suggest that our framework may systematically detect deviation from a particular model assumption and greatly facilitate subsequent model calibration.

## Introduction

Pathogen dynamics are shaped collectively and interdependently by biological processes occurring at the epidemiological, immunological and evolutionary levels. Conventionally, however, each of these processes has been studied independently, revealing only a partial picture of the pathogen dynamics. Phylodynamics studies how these biological processes at various levels act together to shape the phylogeny and transmission of the pathogens [1]. Studies of pathogen phylodynamics are facilitated greatly by increasingly available data sources (particularly, genomic data) [2] and the concurrent development of statistical tools for data integration. In particular, major advances in statistical models that integrate epidemiological and genomic data have been made (e.g., [3–13]). These models have proved very useful for obtaining more comprehensive and detailed pictures of pathogens dynamics− in populations of human, animals and plants. For example, joint epidemiological-evolutionary models have enabled more accurate estimation of transmission histories and a better understanding of the interconnectedness between epidemiological and evolutionary processes.

Despite these advances in model construction and inference, there has been very little development of bespoke diagnostic frameworks for model criticism and, importantly, for *systematically* detecting suspected deviations from particular assumptions in a phylodynamic model in order to guide model refinement. Such a diagnostic framework is crucial given the increasing complexity and diversity of phylodynamic model components and assumptions. In particular, while many phylodynamic frameworks for inferring a transmission history have been proposed [3–5, 7, 10–12, 14–17], various simplifying assumptions made in these models remain to be tested. For example, as it is generally challenging to incorporate and infer within-host-diversity explicitly in a phylodynamic model, many studies assume no pathogen diversity within individual hosts [3, 5, 7, 14–17]. It is often assumed that, within a host, there is a single, dominant pathogen strain at any time *t* and potential within-host-diversity is thereby ignored. Within-host evolution is a known phenomenon for many pathogens (e.g., foot-and-mouth, HIV, Ebola and influenza [18–21]), so that the appropriateness of the single-dominant-strain (s-d-s) approximation in any given scenario should be assessed.

We propose a framework for answering the following questions: (1) *How can we quantify the evidence against model assumptions in a phylodynamic model?*, (2) *If strong evidence is observed, can we discern the nature of model mis-specification so that a more adequate model may be proposed?* Such a framework will greatly facilitate model criticism, refinement and calibration. The notion of using Bayesian *latent residuals* to assess mis-specifications in a spatio-temporal epidemiological model was proposed and exploited in [22, 23]. By proposing appropriately designed *non-centered (re-)parametrizations* of the underlying process, in [22] the authors construct latent residuals whose prior sampling properties are known, and whose posterior samples are sensitive to mis-specifications of the components of a general spatio-temporal epidemiological model. Inferred samples from the posterior distributions of the latent residuals are then assessed against their known sampling distributions, quantifying evidence against model assumptions. The ‘latent–residuals’ approach complements established model-testing tools (e.g., the DIC [24]), allowing diagnostics to be targetted at particular aspects of model formulation. Moreover, for spatio-temporal dynamic models of infectious diseases, it may offer a more sensitive test and more interpretable diagnostics [22, 25].

In this paper, we innovate a model-diagnostic framework for phylodynamic models, utilising and extending the idea of latent residuals. First, we outline generally how latent residuals may be tailored to quantify the evidence against model assumptions in a joint epidemiological-evolutionary spatio-temporal model. Then, we introduce the idea of *marked latent residuals* where we associate an epidemiological quantity (or ‘mark’) with each residual. The marks may then be used to specify subsets of residuals that may potentially be most informative regarding particular mis-specifications of the evolutionary process. Specifically, using simulated data, we show how the marked latent residuals can be used to identify parts of the phylogenetic/epidemic trajectory where modelling assumptions may respectively under- or over-estimate the importance of the within-host evolution of the pathogen. We then apply our diagnostic framework to data describing a localised foot-and-mouth outbreak in the UK, conclusively highlighting the importance of within-host-diversity in modelling the outbreak. In parallel with the model assessment work, we propose a more general model, with an associated pseudo-likelihood, to represent within-host-diversity and significantly improve model fit.

## Models and methods

In this section we give details of phylodynamic models we use in the paper, how these are fitted to data, and the construction of the latent residual process used to assess the quality of model fit.

### The null model *M*_0_: A joint epidemiological-evolutionary spatio-temporal model

#### Epidemic process

We model the epidemic process with a general spatio-temporal stochastic SEIR epidemic model with susceptible (S), exposed (E), infectious (I) and removed (R) compartments. An individual *j* becomes infected (exposed) via background infection with rate *α* and from an infectious individual *i* with rate *βK*(*d*_*ij*_;*κ*). *K*(*d*_*ij*_;*κ*) is the spatial kernel function which characterizes the spatially-dependent infectious challenge from infective *i* to susceptible *j* as a function of distance between them *d*_*ij*_ [26, 27]. Here, we assume *K*(*d*_*ij*_;*κ*) = exp(−*κd*_*ij*_). We use a *Gamma*(*a*, *b*) distribution parameterized by the shape *a* and scale *b* to model the time spent in class *E* (i.e. the latent period), and a *Weibull*(*c*, *d*) parameterized by the shape *c* and scale *d* to model the time spent in class *I* (i.e. the infectious period). In applying to the FMD data, we use *Exponential*(*μ*) for the infectious period, for matching the assumptions of [3, 5]. In simulation studies, we assume *α* = 4 × 10^−4^, *β* = 8, *a* = 8, *b* = 0.5, *c* = 2, *d* = 2 and *κ* = 0.02.

#### Molecular evolutionary process

The molecular evolutionary process of the pathogen is modelled at the level of nucleotide substitutions and is assumed to be conditionally independent of the epidemic parameters given the complete set of epidemic events. This, in effect, means that that genetic evolution does not influence the epidemic parameters so that, for example, there is no selection of increasingly virulent strains. A nucleotide sequence is assembled from bases belong to *purines* (e.g., adenine (*A*) and guanine (*G*)) and *pyrimidines* (e.g., thymine (*U*) and cytosine (*C*)). Substitution between bases in the same category is called *transition* and the substitution between bases from different categories is called *transversion*. Nucleotide bases at different positions of a sequence are assumed to evolve independently according to a continuous-time Markov process. Specifically we use the two-parameter *Kimura model* (Ref. [28]) which allows for different rates of transition and transversion. Under the Kimura model, a nucleotide base *x* mutates to a different nucleotide base *y* within an interval of arbitrary length △*t* with the probability described by Eq 3. We assume that there is a single dominating strain (s-d-s) at each infectious individual at any time point. Upon infection, a newly infected individual is infected with the s-d-s from the source individual. This assumption is consistent with the assumption of no within-host diversity made by other authors [3, 5, 7, 14–17]. In simulation studies, we assume *μ*_1_ = 1 × 10^−4^ and *μ*_2_ = 5 × 10^−5^ where *μ*_1_ and *μ*_2_ represent mutation rates for *transition* and *transversion* respectively.

### A phylodynamic model *M*_1_ for simulating within-host-diversity

In order to assess the effectiveness of our methods we require a mechanism for generating epidemics where within-host diversity is present in the pathogen population. We consider a within-host-diversity model ***M***_1_ with the same epidemic process component used in ***M***_0_. The molecular evolutionary process in each host is described by a continuous-time birth-death process which governs pathogen population growth and death, with mutation occurring along branches. Denote by *N*_*t*_ the current total pathogen population size (of all existing strains) in an infected host. The population size of any strain grows at rate *ν*. We assume there is an equilibrium population size *N*_*e*_ in a host − such an assumption may reflect the fact that there is competition between strains for limited resources in the host [29, 30]. Death occurs at a rate *ν* × *N*_*t*_/*N*_*e*_, so that the equilibrium population size *N*_*e*_ is reached and maintained [29, 30]. Mutations arise at rate *ω* × *N*_*t*_, where *ω* is per-pathogen mutation rate without distinguishing *transition* and *transversion*. An event occurs (i.e., birth, death or mutation) according to their relative rates, and a strain is randomly chosen to experience this event, according to the current population sizes of existing strains. Mutation on the chosen (ancestor) strain creates a new strain (with initial population 1). The new strain has one (randomly chosen) nucleotide position that is different to the ancestor strain. Upon infection, *N*_*B*_ pathogens are randomly chosen (i.e. *N*_*B*_ is the transmission bottleneck) and may be transmitted into the newly infected host in which the pathogen population undergoes the birth-death-mutation process just described. We assume *N*_*e*_ = 3000, *ν* = 3, *ω* = 0.08 and *N*_*B*_ = 200.

### Latent residuals construction for molecular evolutionary process

#### General framework

The construction of latent residuals has its roots in a simple, frequently applied idea which can be illustrated by the following example. Suppose we observe a random sample of observations *y*_1_, …, *y*_*n*_ believed to arise from a continuous distribution with distribution function *F*_*Y*_(*y*; *θ*). Then, using the standard tool of inversion of the distribution function, we can consider each $y_{i}=F_{Y}^{-1}\left(q_{i},\theta \right)$ where *q*_*i*_ is the quantile associated with *y*_*i*_ and, accordingly, *q*_1_, …, *q*_*n*_ is a random sample from a *Unif*(0, 1) distribution. One can then test the fit of the observed *y*_1_, …, *y*_*n*_ to the model *F*_*Y*_ by assessing the fit of *q*_1_, …, *q*_*n*_ to the *Unif*(0, 1) distribution. These quantiles then represent a set of residuals whose sampling distribution is not dependent on *F*_*Y*_ or any model parameter *θ*. Our constructions utilise this basic idea—suitably adapted to accommodate the discrete outcomes, unobserved processes, and parameter uncertainty inherent in the epidemic setting—and embed it within a Bayesian framework. Moreover, we exploit the fact that residuals can be designed, and tested, in a multiplicity of ways to obtain tests targeted at suspected forms of mis-specification.

In the epidemic setting, let ***z*** denote the complete set of events (unobserved and observed) randomly generated from a phylodynamic model ***M*** parameterized by ***θ***. Then, as long as the sampling properties of the phylodynamic model are preserved, we can consider ***z*** to be generated in non-unique ways. In particular, we consider ***z*** as a deterministic function $h_{M,\theta }\left(\tilde{r}\right)$ where $\tilde{r}$ is a random sample from a known distribution, and plays the role of a latent residual process. This representation is essentially a *functional model* as in [31], and exemplifies the concept of generalised residuals proposed in [32]. The process of inversion of the distribution function outlined above provides a simple example of a functional model. Symbolically, we have

$$z=h_{M,\theta }\left(\tilde{r}\right).$$ (1)

Note that, for any ***M***, the selection of a residual process $\tilde{r}$ and a function *h*_***M***,***θ***_(.), can be effected in a multiplicity of ways − and can be tailored to be sensitive to a suspected mode of mis-specification.

In a Bayesian data-augmentation framework, given a random draw (***θ***′, ***z***′) from the posterior distribution *π*(***θ***, ***z***|***y***) (where ***y*** denotes the observed data) it is generally straightforward to invert Eq 1 to impute the corresponding residual $\tilde{r^{\prime }}$ by sampling it from the set $h_{M,\theta }^{-1}\left(z^{\prime }\right)$, the set of residual vectors mapped to ***z***′ by *h*_***M***,***θ***_. Under the hypothesis that the fitted model is correct, then *a priori* $\tilde{r}$ follows the known distribution. We may therefore apply a classical test for consistency with the theoretical distribution to the imputed $\tilde{r^{\prime }}$ (e.g., *Anderson-Darling* hypothesis test [33]) and obtain a posterior distribution of *p-values* $\pi (P\left(\tilde{r}\right)|y)$, summarizing evidence against the modelling assumptions. This distribution represents the posterior distribution of a p-value obtained by a classical observer of $\tilde{r^{\prime }}$ who tests its compliance with its assumed distribution. Should this posterior distribution place high probability on the p-value taking small values, then with high posterior probability the classical observer would reject the hypothesised model for $\tilde{r}$. The general approach is discussed in more detail in [34].

The latent–residual approach extends the ideas underlying posterior predictive checking [35] and can be viewed in that general context. We note that posterior predictive checking has been previously applied in the phylogenetic setting. For example, in [36] the fit of a phylogenetic model is assessed by comparing clustering properties of observed trees with the distribution of clustering properties on trees simulated from the posterior predictive distribution. Our approach builds on the standard approach to posterior predictive checking through its use of discrepency variables defined in terms of imputed, rather than directly observed processes, exploiting the freedom afforded by the Bayesian approach to tailor the imputed latent processes so that tests can be targetted at specific modes of model inadequacy. A further difference lies in the use of the full posterior distribution of the resulting imputed p-value to summarise evidence against the model—rather than its expectation as expressed by the usual posterior predictive p-value. Finally we note that, by imputing quantities with a sampling distribution that is fixed under the assumed model and independent of model parameters, we dispense with any need to simulate from their posterior predictive distribution.

#### Marked latent residuals for model *M*_0_

Given a realization of the epidemic process, consider a pair of pathogen sequences *G*_*A*_ and *G*_*B*_ on an infected host at *consecutive ‘critical time points’* *t*_*A*_ < *t*_*B*_ (where a critical point is a transmission or a (sequence) sampling event). Assuming *G*_*A*_ evolves to *G*_*B*_ during the interval (*t*_*A*_, *t*_*B*_) according to the continuous-time Markov process specified in ***M***_0_, the number of observed mutations (i.e. change of nucleotide bases) among *n* nucleotides on *G*_*A*_ is distributed as

$$m∼Bin(n,p_{△t})$$ (2)

where △*t* = *t*_*B*_ − *t*_*A*_ and

$$p_{△t}=0.75-0.25e^{-4\mu_{2}△t}-0.5e^{-2(\mu_{1}+\mu_{2})△t}.$$ (3)

and *μ*_1_ and *μ*_2_ are the rates of *transition* and *transversion* respectively.

We now design a process $\tilde{r}$ and a function $h_{M_{0}}\left(.\right)$ in order that the statistical test on $\tilde{r}$ be sensitive to deviation from the s-d-s assumption that underpins molecular evolution. Specifically, we formulate a functional model for the molecular evolutionary process in which components of $\tilde{r}$ are independently distributed as

$$\tilde{r}∼Unif\left(0,1\right).$$ (4)

The process described by Eq 2 may be reconstructed as

$$m=\inf\{\;m^{\prime }\;|\;\sum_{k=0}^{m^{\prime }}q_{\left(k\right)}>\tilde{r}\},$$ (5)

where *q*_(.)_ is the cumulative distribution function of the Binomial distribution in Eq 2. Note that this formulation specifies only the numbers of mutations occurring within a time window; the specific sites at which mutations occur are not specified by $\tilde{r}$.

**Fig 1:**
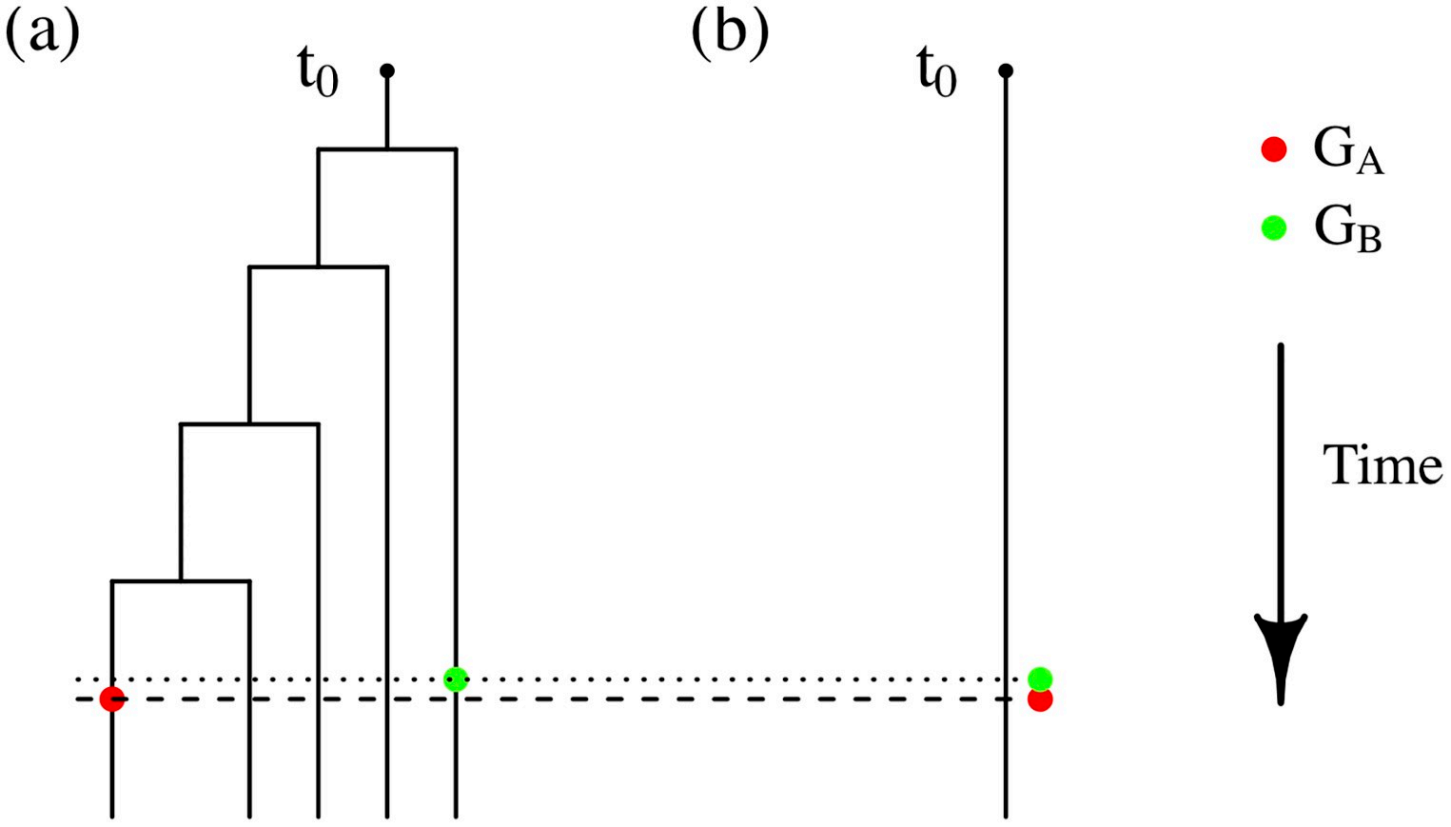
Schematic illustration of the rationale of the marked latent residuals. Assume that a single pathogen strain enters a host and begins to evolve at time *t*_0_. A within-host-diversity model, crudely illustrated by (a), allows for establishment of new strains (i.e. new branches) generated from mutations (occurred at internal nodes). The s-d-s model ***M***_0_, illustrated by (b), allows only mutations along the (linear) line/branch and as a result assumes only one (dominant) strain at any particular time point. Assume that two sequence samples *G*_*A*_ and *G*_*B*_ (superscripts dropped without ambiguity) are randomly sampled from the pathogen population at *t*_*A*_ and *t*_*B*_ respectively, where *t*_*A*_ − *t*_*B*_ ≈ 0 (i.e. *ζ*^(*k*)^ ≈ 1). (a) may predict distinct *G*_*A*_ and *G*_*B*_, while (b) would predict minimal difference between them due to the implied linear relationship between mutations and time. Therefore, residuals associated with high *ζ*^(*k*)^ may be expected to be large if a model takes insufficient account of within-host-diversity.

The residuals in $\tilde{r}$ can be further associated with specific quantities or ‘marks’ characterized by the realized epidemic process. Let $t_{A}^{\left(k\right)}$ and $t_{B}^{\left(k\right)}$ denote the corresponding critical time points for the *k*^*th*^ pair of consecutive sequences $G_{A}^{\left(k\right)}$ and $G_{B}^{\left(k\right)}$ sampled (or imputed) from a particular infected host. The corresponding residual $\tilde{r}$ is then associated with the *mark*

$$\zeta^{\left(k\right)}=\frac{t_{A}^{\left(k\right)}-t_{0}}{t_{B}^{\left(k\right)}-t_{0}}\in \left(0,1\right),$$ (6)

where *t*_0_ is the time of infection of the host. Note that these marks are determined solely by the epidemic process and that the residual associated with a mark is therefore independent of the value of the mark in our functional-model representation. The s-d-s model ***M***_0_ assumes a relationship between the expected number of mutations (effectively a ‘genetic’ time) and the time difference *t*_*B*_ − *t*_*A*_ that is approximately linear when this difference is small. The quantity *ζ*^(*k*)^ is designed to identify situations where − proportionately − the ‘effective’ genetic time between $G_{A}^{\left(k\right)}$ and $G_{B}^{\left(k\right)}$ might deviate from that predicted by ***M***_0_. Suppose that we fit ***M***_0_ to data generated from a model with considerable within-host-diversity. One may expect that this deviation would be most prominent when *ζ*^(*k*)^ is large (i.e. when $t_{A}^{\left(k\right)}\approx t_{B}^{\left(k\right)}$ and *ζ*^(*k*)^ ≈ 1). As $t_{A}^{\left(k\right)}-t_{B}^{\left(k\right)}\approx 0$, ***M***_0_ would predict very few mutations between $G_{A}^{\left(k\right)}$ and $G_{B}^{\left(k\right)}$, while the within-host-diversity may lead to a substantial difference between $G_{A}^{\left(k\right)}$ and $G_{B}^{\left(k\right)}$. Fig 1 illustrates schematically the rationale behind the marked latent residuals. Therefore, the deviation from the s-d-s assumption should be *systematically* reflected in the distribution of the imputed residuals associated with large *ζ*^(*k*)^ (see Results). In particular, according to (the inversion of) Eq 5, we expect to observe a concentration of (imputed) residuals close to 1 when attention is restricted to residuals associated with high mark *ζ*^(*k*)^ (Results). We therefore anticipate, for example, that a test that restricts attention to a subset of residuals with non-zero marks may be more sensitive to within-host pathogen diversity than one based on the full set of residuals (Results).

#### Associating with superspreading events

*Superspreading* is a common phenomenon in many infectious disease epidemics [26, 37] in which numerous infections by a given host occur within a relatively short period of time and multiple pathogen-sequence pairs are sampled/transmitted closely in time (see Fig 1). Since systematic deviation from *Unif*(0, 1) is expected in residuals associated with high marks, and high marks may be more numerous in the event of superspreading, one may naturally conjecture that deviations from the s-d-s model, as detected using our methods, may be most apparent when superspreading occurs.

The general ideas underpinning the construction of latent residuals can be applied to design tests to detect other modes of mis-specification. The imputation of ‘infection-link’ residuals to detect mis-specification of spatial kernel functions has been described in [22], where residuals to detect mis-specification of sojourn time distributions are also considered. The construction of the marked genetic residuals described here could also be modified to detect alternative suspected modes of mis-specification of a genetic model. The key aim would be to identify imputable outcomes from the epidemic model (e.g. exposure times of individuals, properties of the transmission graph) that can be used to specify marks in such a way that an association between marks and residuals would be expected should the suspected mis-specification be present. For example, were it suspected that the mutation rate of the pathogen population were increasing or decreasing over time, then we might redefine the mark associated with a given residual (see Eq (6)) to simply be *t*_*A*_—the infection time of the source imputed for a particular infection event. Any increase in the true mutation rate over time should then induce some systematic dependence of imputed residuals on the corresponding mark, with small (resp. high) marks tending to be associated with small (resp. high) residuals. Were it suspected that the pathogen’s ability to reproduce itself tended to be less in the recipient than in the donor of infection, then this deviation may be detected by defining the mark associated a residual to be the depth of the corresponding donor in the imputed transmission graph, and testing for an association between residuals and the corresponding mark. Either of these tests could be implemented via comparatively minor modifications of the algorithms presented here.

### Statistical inference

An overview of the Bayesian inference procedures used in the paper is given in *SI: SI Text*. In summary, given observations *y*, we use standard Bayesian data-augmentation approaches to generate samples from *π*(***θ***, ***z*** = (***z***_*e*_, ***z***_*g*_)|***y***) where *z*_*e*_ comprises the times and nature of all transitions occurring during the epidemic and *z*_*g*_ comprises the set of observed genetic sequences augmented with the set of sequences passed during transmission events. These techniques, as applied to the model ***M***_0_, are described in detail in [3].

Given a draw from *π*(***θ***, ***z*** = (***z***_*e*_, ***z***_*g*_)|***y***), it is straightforward to impute the corresponding latent residual process $\tilde{r^{\prime }}$ by reversing the procedure described in Eq 5, given *m* and the other model parameters inferred in the Bayesian framework. The imputed $\tilde{r^{\prime }}$ is then compared to its sampling distribution (i.i.d. *Unif*(0, 1)) to quantify the evidence against model ***M***_0_ −here using the posterior distribution of the associated p-value, $\pi (P\left(\tilde{r}\right)|y)$. When strong evidence against the model is observed, the corresponding marked latent residuals may be inspected to elicit the nature of the poor fit (Results).

#### Model refinement: Generalizing the S-D-S model

While it may be straightforward to simulate data-sets using Model ***M***_1_ inference with this model, using the approach applied to Model ***M***_0_, is problematic due to intractability of the genetic component of the likelihood. We therefore propose an alternative inferential framework—effectively using a surrogate model—that can represents within-host-diversity and attempt to assess its adequacy using our methods.

Assume that an infectious individual infects individuals at times *t*_1_ < *t*_2_ < … during the period [*t*_0_, *t*_*f*_]. The s-d-s model ***M***_0_ assumes that the strain transmitted at *t*_*k*_ is a direct descendent of that transmitted at *t*_*k*−1_ and a full likelihood function can be constructed for the genetic differences between strains assuming a comparatively simple mutation model [3]. However, while it is possible to simulate within-host evolution from a mechanistic within-host-diversity model (e.g., from model ***M***_1_, see Models), it may not be straightforward to perform inference with the dynamical model used. We therefore formulate a pseudo-likelihood framework ***M***_*p*_ which takes into account within-host-diversity (and allows departure from the s-d-s assumption), and includes the s-d-s model ***M***_0_ as a limiting case.

We introduce a framework which represents an ‘effective genetic time difference’ between two strains randomly chosen from the population within a host (and transmitted) at critical time points *t*_*A*_, *t*_*B*_ ∈ [*t*_0_, *t*_*f*_]. The effective time difference between the two strains *G*_*A*_ and *G*_*B*_, transmitted at times *t*_*A*_ < *t*_*B*_, is defined to be:

$$T\left(G_{A},G_{B}\right)=t_{A}+t_{B}-2T_{c}\left(G_{A},G_{B}\right)$$ (7)

where *t*_*A*_ ≥ *T*_*c*_(*G*_*A*_, *G*_*B*_) ≥ *t*_0_ is the latest time up to which ancestry of *G*_*A*_ and *G*_*B*_ is common. Now *T*(*G*_*A*_, *G*_*B*_) is unknown so we treat it as a random variable. A mutation event (i.e. a nucleotide at a same position on *G*_*A*_ and *G*_*B*_ being different) may be then described by a simple probabilistic model, i.e., probability of a mutation

$$p_{T(G_{A},G_{B})}=1-e^{-\lambda T(G_{A},G_{B})}$$ (8)

where λ represents a mutation rate (note that we use single parameter λ for mutation rate as opposed to the two-parameter setting in the s-d-s model). Note that *t*_*B*_ − *t*_*A*_ < *T*(*G*_*A*_, *G*_*B*_) < *t*_*B*_ + *t*_*A*_ − 2*t*_0_. Under our approach we assume

$$T^{*}\left(G_{A},G_{B}\right)∼\text{Beta}\left(\gamma ,\eta \right),$$ (9)

where

$$T^{*}\left(G_{A},G_{B}\right)=\frac{T\left(G_{A},G_{B}\right)-t_{B}+t_{A}}{2(t_{A}-t_{0})}\in \left(0,1\right).$$ (10)

We then formulate a ‘pseudo-likelihood’ function for the complete genetic data by augmenting the genetic data with the unknown *T*(*G*_*A*_, *G*_*B*_) (or equivalently T*(*G*_*A*_, *G*_*B*_)) for each successive pair of transmitted strains, by assuming that the relationship of *G*_*B*_ to *G*_*A*_ is independent of the latter’s relationship to any previously sampled or transmitted strain strains. Details of the formulation of the likelihood function are given in *SI*: S1 Text. Note that the s-d-s model would arise as a special case of the above in the limit where *γ* is fixed and *η* → ∞. The corresponding limiting distribution for T*(*G*_*A*_, *G*_*B*_) places unit probability on T*(*G*_*A*_, *G*_*B*_) = 0, so that the effective time difference reduces to *t*_*B*_ − *t*_*A*_. Model inference is performed by adapting the MCMC algorithm used to fit the s-d-s model in [3] by further augmenting the parameter vector with the T*(*G*_*A*_, *G*_*B*_) and replacing the part of the likelihood contributed by the evolutionary process with a pseudo-likelihood function (as detailed in *SI*: S1 Text).

Latent residuals under this pseudo-likelihood framework ***M***_*p*_ may be constructed in a fashion similar to that applied to ***M***_0_, by replacing the probability of observing a mutation *p*_Δ*t*_ (under the s-d-s assumption) in Eq 3 by *p*_*T*(*G*_*A*_, *G*_*B*_)_ (which considers within-host diversity) in Eq 8.

Although we have a simple Jukes-Cantor substitution model in ***M***_*p*_, in the interests of reducing complexity, we remark that the approach can be applied, with little modification, when the Kimura model is used in ***M***_*p*_, since the probability of mutation at a site in a given interval is not dependent on the base at the site in question. Hence the number of mutations in a given interval follows a binomial distribution and a residual can be imputed based on the quantile function of the binomial, as we do here. Note that we could define a second residual process related to the relative frequency of transitions and transversions by first noting that, conditional on the number of of mutations being *m*, the number of transitions follows a *Bin*(*m*, *p*) distribution for some appropriate *p* calculable from the generator matrix of the continuous-time Markov process defining the dynamics of mutations at a site, and the effective time *T*(*G*_*A*_, *G*_*B*_). Intuitively, we may expect evidence against the model’s assumptions on within-host diversity to be most apparent from inspection of the first set of residuals. Accordingly, we may only impute, and test against the assumption of uniformity, the first residual process relating to the number of mutations. For the Jukes-Cantor model we remark that $p=\frac{1}{3}$. Hence, if we wished to test our Jukes-Kantor assumption using this framework we may consider the second residual process, imputed under the Jukes-Kantor assumption $p=\frac{1}{3}$ and test for deviations from *U*(0, 1). Were a more complex substitution model, incorporating base-dependent transition rates, then functional-model representations—along with related residual processes—could nevertheless be constructed, by representing mutations between strains *G*_*A*_ and *G*_*B*_ in a given effective period in terms of four distinct, independent binomial distributions with parameters (*n*_*A*_, *p*_*A*_), (*n*_*T*_, *p*_*T*_), (*n*_*G*_, *p*_*G*_), (*n*_*C*_, *p*_*C*_) where the first parameters denote the numbers of sites occupied by each of the respective four bases and the second parameters denote the respective mutation probabilities. We therefore believe that our basic approach can be tailored to settings where the mutation process at sites follows a general continuous-time Markov process.

## Results

### Simulation studies

To test our diagnostic framework, we consider two scenarios: (I) the s-d-s (null) model ***M***_0_ is fitted to data generated from ***M***_0_ itself and (II) ***M***_0_ is (inappropriately) fitted to data generated from a within-host-diversity model ***M***_1_. We use the same (SEIR) spatio-temporal epidemic process component in both ***M***_0_ and ***M***_1_. Instead of assuming s-d-s, ***M***_1_ embodies a continuous-time evolutionary process that accounts for growth, death and mutation of pathogen strains. Details of the models are given in *Models*.

We first simulate an epidemiological dataset ***z***_*e*_ among a susceptible population (with size *N* = 150, generated as a random sample from a uniform distribution over a square region) using the common epidemic process component shared by ***M***_0_ and ***M***_1_, during a period (0, *T*_*max*_). Data ***z***_*e*_ comprise typical epidemiological events, including infection time, transition times from compartment E to I and from I to R and transmission path. Conditioning on this ***z***_*e*_, several sets of (different) sequence data ***z***_*g*_ are then simulated, using the evolutionary component (s-d-s) in ***M***_0_ (Scenario I) in a single case and that in ***M***_1_ (Scenario II) in 5 cases. Using the same set of ***z***_*e*_ (and epidemic process component) ensures that any discrepancy in the evidence of mis-specification between the two scenarios arises from the difference of the evolutionary component.

In each scenario, we use Bayesian data augmentation to generate a sample from *π*(***θ***, ***z*** = (***z***_*e*_, ***z***_*g*_)|***y***) from which we impute $\tilde{r^{\prime }}$, from the posterior distribution of the residual vector (*SI*: S1 Text.) Here the observed data ***y*** include: times and locations of all transitions from E to I and from I to R, and sequences sampled for each infected host at a random sampling time. Transmission path, infection times and sequences transmitted during infection events are assumed to be unknown.

**Fig 2:**
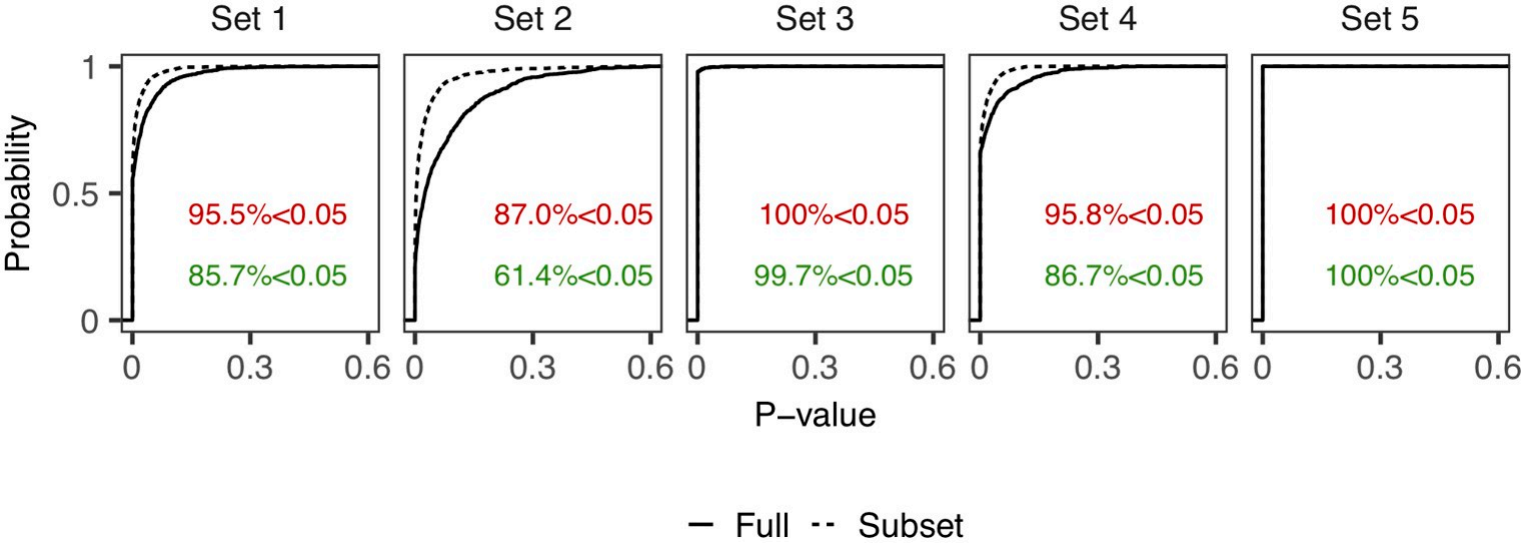
Empirical cumulative distribution functions of p-value obtained by applying the Anderson-Darling test to the subset of residuals with non-zero marks and to the full set of residuals (for simulations set 1 to set 5 in Scenario II where s-d-s model *M*_0_ is (inappropriately) fitted to data generated from a within-host-diversity model *M*_1_), see also Table 1. Proportions of p-value less than 0.05 (indicated by colored text) in this case are consistently higher for the subset of residuals corresponding to non-zero marks (red text).

For the assumed model ***M***_0_ *a priori* $\tilde{r}$ is distributed as a random sample from a *Unif*(0, 1). Moreover, if ${\tilde{r}}^{*}$ denotes the subset of $\tilde{r}$ with non-zero associated marks, then *a priori* ${\tilde{r}}^{*}$ is also a random sample from *Unif*(0, 1). Then we apply an *Anderson-Darling* test to $\tilde{r}$, and to the subset ${\tilde{r}}^{*}$ to test for consistency with the uniform distribution. We can summarize the evidence against model assumptions from the posterior distribution of the p-values using summary statistics such as $\pi (P\left(\tilde{r}\right)<0.05|y)$ (mainly used here) as well as the empirical distribution functions of the p-values. Table 1 shows clear evidence against the null model ***M***_0_ in scenario II. Fig 2 shows that by using the subset ${\tilde{r}}^{*}$ we consistently obtain more evidence against the s-d-s model, notably in the case Set 2 where the conclusion is relatively ambiguous.

**Table 1:** Proportions of p-value less than 0.05 that indicate overall evidence against the null model in two scenarios: (1) fitting the correct model structures and (2) fitting the s-d-s *M*_0_ to data generated from a within-host-diversity model *M*_1_. For scenario (2), five datasets are generated independently from ***M***_1_ with a same set of parameter values (*Models*), which are used to reveal any consistent difference of the evidence of model mis-specification compared to scenario (1). Noted that in both scenarios, the (same) correct epidemic process component is fitted. ${\tilde{r}}^{*}$ is the subset of the full set of residuals $\tilde{r}$, associated with non-zero marks *ζ*^*k*^.

Model Fitted	$\pi (P\left(\tilde{r}\right)<0.05|y)$	$\pi (P\left({\tilde{r}}^{*}\right)<0.05|y)$
***M***_0_	4.7%	4.4%
***M***_1_ (Set 1)	85.7%	95.5%
***M***_1_ (Set 2)	61.4%	87.0%
***M***_1_ (Set 3)	99.7%	100%
***M***_1_ (Set 4)	86.7%	95.8%
***M***_1_ (Set 5)	100%	100%

**Fig 3:**
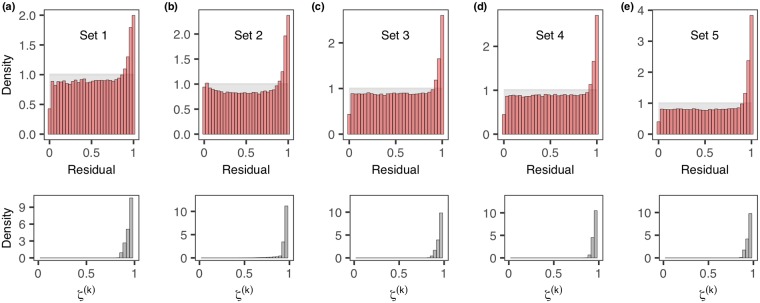
Systematic deviation revealed by the marked latent residuals. (a)-(e) correspond to simulation set 1-5 (from Scenario II where s-d-s model ***M***_0_ is (inappropriately) fitted to data generated from a within-host-diversity model ***M***_1_). The histograms depicted in the first row are formed by aggregating residuals whose associated marks *ζ*^(*k*)^ lie in the top tercile of marks for any posterior sample for which the p-value of the Anderson-Darling test is less than 0.05. The histograms of *ζ*^(*k*)^ are shown in the second row. Residuals associated with smaller *ζ*^(*k*)^ may exhibit a multiplicity of patterns (see *S*I: S1 Fig).

Having observed strong evidence against the null model in scenario II, we investigate the imputed residuals conditional on the p-value being less than 0.05 to detect any systematic pattern. Fig 3 shows that, as conjectured, for *ζ*^(*k*)^ with values in the upper tercile of the set of marks, the corresponding residuals are consistently disproportionately located at the right-hand end of the interval (0, 1), suggesting that model ***M***_0_ may take insufficient account of within-host-diversity leading to the poor model-fit suggested by Table 1.

Also, S2 Fig in *SI* shows that inferred posterior distributions can accurately recover the true values of the model parameters when fitting the correct model.

### Case study: Animal foot-and-mouth disease outbreak

#### Model diagnosis

In this section we apply our diagnostic framework to a localized FMD outbreak in the UK (Darlington, Durham County) in 2001 previously analysed by several authors (e.g., [3, 5]). Some 15 infected premises (i.e. farms, indexed by by the letters A-P) were observed, from which one virus sequence for each premises with sequence length *n* = 8176 was sampled [5, 13]. The geographical locations, the removal (i.e. culling) times and the genome sampling times of the infected premises were also reported. Here we fit the model ***M***_0_ (also fitted in [3]) and use our diagnostic framework to elicit evidence of mis-specification.

Despite the relatively small size of this dataset, our residuals detect notable evidence against the model ***M***_0_ both using $\tilde{r}$ and ${\tilde{r}}^{*}$. Specifically we find that $\pi (P\left(\tilde{r}\right)<0.05|y)$ = 100% and $\pi (P\left({\tilde{r}}^{*}\right)<0.05|y)$ = 80%. The corresponding proportions become respectively 100% and 61% when the significance level 0.05 is replaced by the more conservative 0.01.

**Fig 4:**
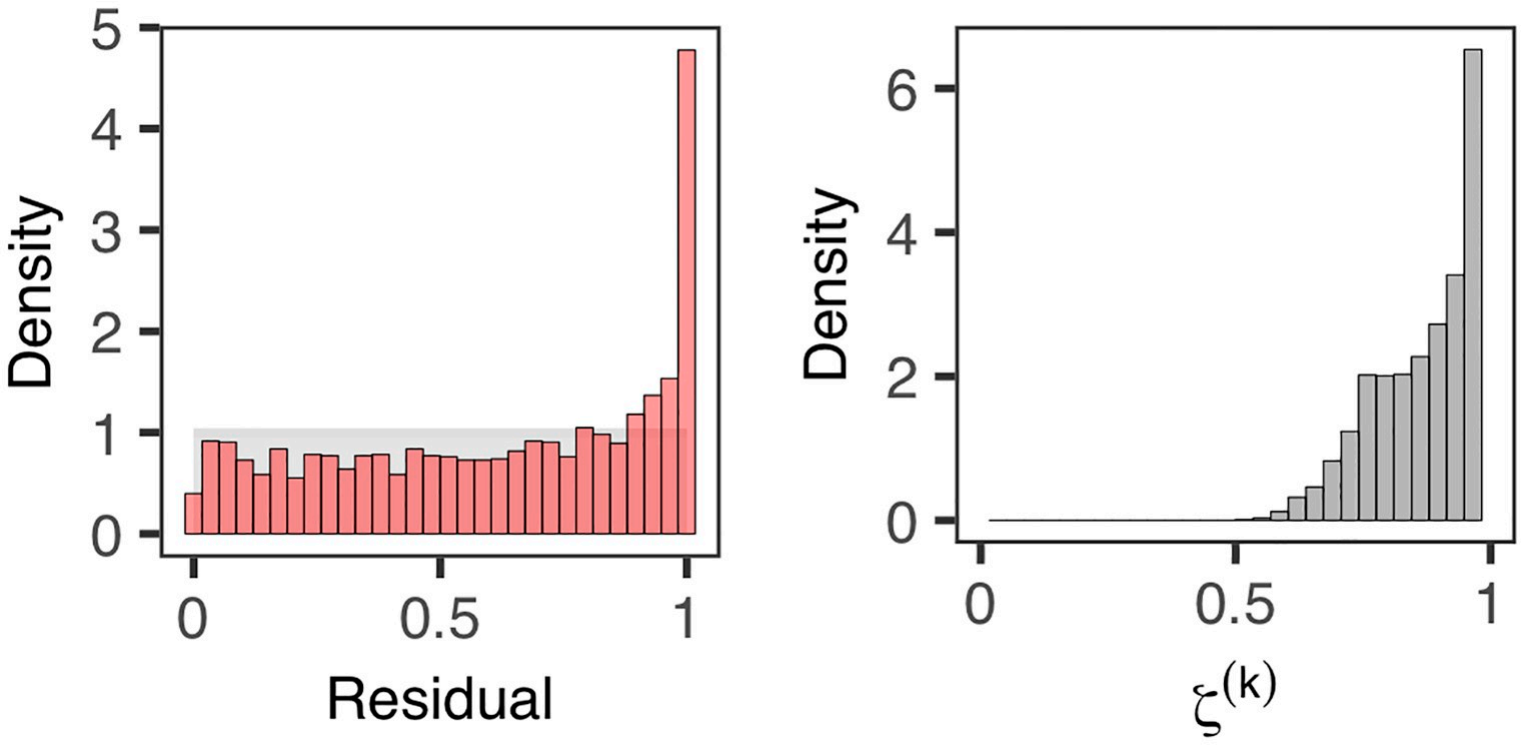
Residuals associated with the top tercile of marks *ζ*^(*k*)^, in applying our diagnostic framework to a foot-and-mouth dataset.

Fig 4 further reveals that within-host-diversity has been considerably under-estimated by fitting ***M***_0_. It is observed that, in this case, the subset of residuals ${\tilde{r}}^{*}$ yields less evidence than $\tilde{r}$, plausibly due to the the small outbreak size, the small sample size of ${\tilde{r}}^{*}$ and the reduced potential for superspreading events. We observed that few marks attain higher values close to unity (Fig 4) compared to the simulated scenarios (Fig 3) where the outbreak size (*N* = 150) is much larger. The results are also consistent with studies of sequence diversity of FMD virus (e.g. [18]) suggesting considerable within-host-diversity for FMD virus.

**Fig 5:**
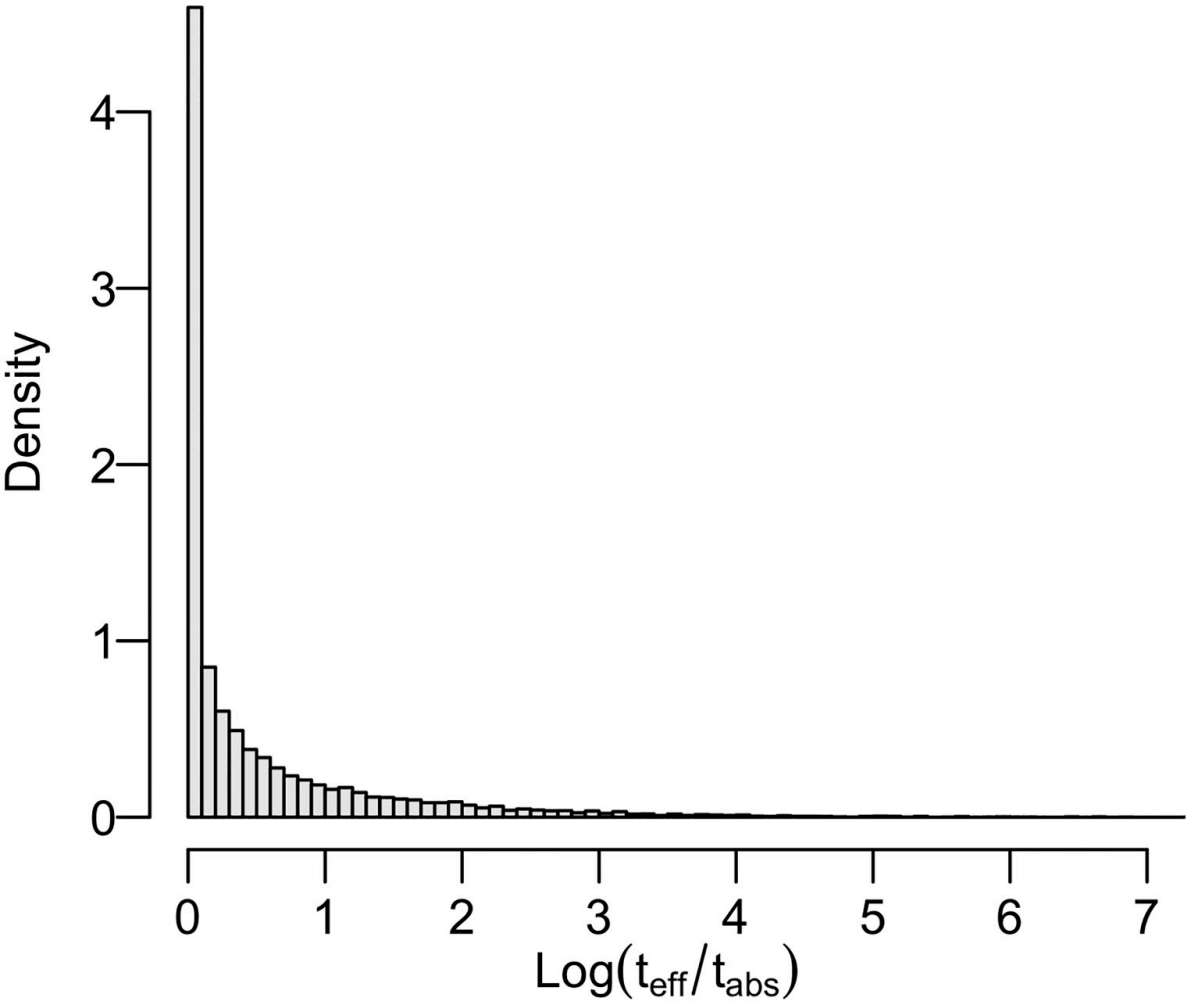
Logarithm of the ratio between inferred effective genetic time *t*_*eff*_ = *T*(*G*_*A*_, *G*_*B*_) and the ‘absolute’ genetic time *t*_*abs*_ = *t*_*B*_ − *t*_*A*_ used in the s-d-s model. Consider an individual who becomes infected at time *t*_0_ = 0 and causes two infections at times *t*_*A*_ and *t*_*B*_ where *t*_*A*_ < *t*_*B*_ are generated as the first two event times in a Poisson process. Then we have *t*_*eff*_/*t*_*abs*_ = 1 + 2 × T*(*G*_*A*_, *G*_*B*_) × *Z* (see also Eq 10) where *Z* = *u*/(1 − *u*) with *u* ∼ *Unif*(0, 1). For each simulated *Z*, we draw a corresponding T*(*G*_*A*_, *G*_*B*_) from Beta(γ,η) (see Eq 10) where *γ* and *η* are taken to be their respective posterior means.

As our results (Fig 4) reveal considerable evidence against the s-d-s model ***M***_0_, suggesting that it may not take sufficient account of within-host diversity. it is natural to attempt to refine this ‘inadequate’ model and to fit the more general model ***M***_*p*_. There is much weaker evidence against the model when ***M***_*p*_ is fitted to the FMD outbreak using the pseudo-likelihood approach and latent residuals are imputed to yield a distribution of p-values. In particular we obtain $\pi (P\left(\tilde{r}\right)<0.05|y)$ = 43% and $\pi (P\left(\tilde{r}\right)<0.01|y)$ = 14%, which are considerably less than the 100% obtained by applying both metrics on the full set of residuals $\tilde{r}$ in fitting the s-d-s model ***M***_0_. This result reinforces the conclusion that the s-d-s assumption may be one root of model mis-specification (Fig 4(b)), and suggests that including within-host diversity may serve to increase model adequacy. Fig 5 shows that the effective genetic time *T*(*G*_*A*_, *G*_*B*_) may be considerably larger than the ‘absolute’ genetic time *t*_*B*_ − *t*_*A*_ used in the s-d-s model, given our estimated $T^{*}\left(G_{A},G_{B}\right)∼\text{Beta}\left(\gamma ,\eta \right)$ (see also Eq 10). It is worth noting that in using the pseudo-likelihood framework to fit ***M***_*p*_ we obtained a smaller mean mutation rate 4.32 × 10^−5^, when compared to the case of fitting the s-d-s model in which we had an overall mean mutation rate (including transition and transversion) 6.41 × 10^−5^. Our results suggest that observed amount of mutations in FMD may be better explained by a combination of a smaller mutation rate and a longer effective genetic time that takes into account the within-host diversity, as opposed to a larger mutation rate with a shorter absolute genetic time as implied by the s-d-s model.

An important question is that of whether inferred parameters of the epidemic model, or imputed events in a partially observed epidemic, are sensitive to mis-specification of the genetic model. Therefore in the SI we compare the posterior distributions of epidemic parameters and of imputed infection graphs respectively using the s-d-s assumptions and the pseudo-likelihood framework that takes into account within-host diversity. We note that while estimated values of most key epidemiological parameters appear to be very similar over the two analyses (see *S*I: S3 Fig) the posterior distribution of *β*, the secondary transmission rate, places considerably more weight on higher values in the case of the pseudo-likelihood analysis, pointing to the potential for the s-d-s assumptions to lead to underestimation of this parameter should they be inappropriate. Comparison of the *a posteriori* most probable transmission trees under the two approaches (see *S*I: S4 Fig) suggests that while the trees share many similarities, they differ in their inferences regarding the relative importance of sites A and K as infectors in the epidemic. Under s-d-s assumptions A and K are the sources 2 and 5 infections respectively in the modal infection graph. With the pseudo-likelihood framework these values become 5 and 1 respectively.

## Discussion

Major statistical advances for integrating epidemiological and genomic data have been stimulated and accomplished in recent years, in the midst of ever-increasingly available genomic data (e.g., [3–13]). Given the (increasing) complexity and variety of phylodynamic models, it is crucial to develop model diagnostic methods that may systematically detect specified deviations from particular model assumptions. Such tools would greatly facilitate model criticism, calibration and refinement. While conventional model testing and model selection techniques such as Bayes factors and Deviance Information Criterion (DIC) can be very useful for comparing competing models, they do not offer an interpretable framework that can be used for fine component-wise model diagnostics and refinement [22, 24, 38]. Moreover, in contrast to the latent-residual approach [22], they may be less sensitive in the context of spatio-temporal dynamic modelling of infectious disease [25].

In this paper we have proposed a novel model diagnostic framework that extends the notions of functional models and latent residuals [22, 23] to phylodynamic processes. Our framework can be easily embedded within any Bayesian analysis of a spatio-temporal phylodynamic system that makes use of data-augmentation. Overall evidence against model assumptions is evaluated by assessing sets of latent residuals sampled from the posterior distribution for consistency with the assumed sampling properties of the residuals. We also particularly show that how a marked latent-residual process can be tailored to reveal the nature of mis-specification of the molecular evolution process specified in a phylodynamic model. Using simulated datasets, we exemplify our approach by showing how the marked latent residuals can be used to reveal and quantify the under-estimation of the importance of within-host-diversity in a fitted phylodynamic model. Furthermore, we show that the tailored marked latent residual testing can be particularly powerful in the event of superspreading. Our framework is then applied to a local FMD outbreak in UK, the results suggesting that the importance of within-host-diversity may be considerably under-estimated by the s-d-s models [18]. Finally, we demonstrate that our diagnostic framework could facilitate effective model calibration. Specifically, we propose a pseudo-likelihood framework which allows for a higher degree of within-host-diversity, significantly improving the model adequacy as assessed using the latent-residual approach.

We have considered testing schemes utilising respectively the full set of residuals $\tilde{r}$ and those associated with non-zero marks ${\tilde{r}}^{*}$. The results suggest differences in the strength of evidence against the assumed model provided by these two approaches but that one is not invariably superior to the other. The procedure presented can only be valid if the rule for selecting residuals is specified *a priori*; post-hoc selection of a maximally informative subset is clearly not acceptable. This points to an interesting ‘virtual’ design problem of determining the rule for selecting residuals, based on the Bayesian’s assumed model and parameter prior and a specific sampling distribution for the observations under the suspected mis-specified model, in order to maximise the expectation of some measure of evidence against *M*_0_.

We have exemplified our diagnostic framework by using it to test the appropriateness of the s-d-s assumption. Such a framework is broadly applicable to other phylodynamic systems (e.g., influenza and Ebola [20, 21]) where quantifying importance of within-host diversity in any given scenarios is crucial. In principle, the framework could be extended to test other model assumptions by, for example, formulating different (epidemiological) marks *ζ*^(*k*)^ to associate with the residuals to reflect deviations from other assumptions for the evolutionary process. We claim no optimality of the pseudo-likelihood framework proposed for the FMD which utilises several simplifying assumptions of independence in the evolutionary process. Nevertheless, since our objective is to seek further evidence that the under-estimation of the importance of within-host-diversity is the source of model mis-specification (indicated by Fig 4) by showing improved fit of a model that represents diversity, we believe that its use may be justified.

The results of this paper complement earlier results [3] where bespoke, infection-link residuals were used to detect mis-specification of the spatial kernel in model ***M***_0_ in the setting where both epidemic and genetic information were available. In a simulation study, detailed in the SI of reference [3] (Table S7 therein), it is shown that the evidence against a model with mis-specified kernel provided by the infection-link residuals, appears to be enhanced when genetic information is combined with the epidemic data. Our results here demonstrate the feasibility of using alternatively formulated residuals to detect a different form of mis-specification, specifically the genetic component in model ***M***_0_, providing support for the broad applicability of the general approach. The marked latent residual approach should also be easily scaleable for larger epidemics as the number of residuals only increases linearly with of transmission and sequence sampling events.

## Supporting information

S1 Text Supplementary information. (1) Bayesian data-augmentation and model inference of S-D-S Model ***M***_0_. (2) Refining S-D-S model ***M***_0_.
(PDF)

S1 FigDistributions of subsets of marked latent residuals that lead to p-value less than 0.05.(a)-(e) correspond to simulation set 1-5. Each set of the imputed residuals $\tilde{r^{\prime }}$ are first ordered according to the mark *ζ*^(*k*)^. The ordered residuals are then subdivided into three equal-size samples − 1^*st*^, 2^*nd*^ and 3^*rd*^ one-third. Residuals associated with smaller *ζ*^(*k*)^ (1^*st*^ ans 2^*nd*^) exhibit a multiplicity of patterns, as opposed to the 3^*rd*^ one-third where deviation is consistently observed at the right-tail of the unit interval (0, 1) (see also main text).(TIFF)

S2 Fig Posterior distributions of key model parameters when fitting the correct model *M*_0_ to the simulated dataset. (TIFF)

S3 Fig Posterior distributions of key epidemiological model parameters when fitting respectively the s-d-s model and the pseudo-likelihood model that takes into account within-host diversity to the FMD dataset. (TIFF)

S4 Fig Estimated most probable transmission trees when fitting respectively the s-d-s model and the pseudo-likelihood model that takes into account within-host diversity to the FMD dataset. (a) Obtained from fitting the s-d-s model; (b) Obtained from fitting the pseudo-likelihood model.
(TIFF)

S1 Table Prior distributions for model parameters. (PDF)
