# Supplementary material for: Model diagnostics and refinement for phylodynamic models
Source: PLoS Comput Biol. 2019 Apr 5;15(4):e1006955. doi: 10.1371/journal.pcbi.1006955 (PMC6469796; doi:10.1371/journal.pcbi.1006955)
Supplement: S1 Text — (1) Bayesian data-augmentation and model inference of S-D-S Model M0. (2) Refining S-D-S model M0. (PDF) [file pcbi.1006955.s001.pdf]

Supporting Information

S1 Text

Bayesian Data-augmentation and Model Inference of S-D-S Model $\boldsymbol{M}_0$

$\boldsymbol{M}_0$ was first proposed in [3] in which inference procedures were described in detail. Here we give an overview of the general principles of the model inference. It is now standard practice to conduct Bayesian analyses of partially observed epidemics using the process of data augmentation supported by computational techniques such as Markov chain Monte Carlo methods. Given observed partial data $\boldsymbol{y}$, such as times of symptom onset, these approaches involve sampling from the joint posterior distribution $\pi(\boldsymbol{\theta}, \boldsymbol{z}|\boldsymbol{y})$ where $\boldsymbol{z}$ represents the complete data and $\pi(\boldsymbol{\theta})$ represents the prior distribution of model quantities, such that the complete $\boldsymbol{z}$ is reconstructed, or 'imputed'. In our application, $\boldsymbol{z}$ involves both partially observed epidemic and sequence data. The crucial element in the model inference of $\boldsymbol{M}_0$ is the joint imputation of unobserved sequences, transmission path and unobserved infection times.

The central idea is to jointly propose a new and statistically consistent sequence somewhere between two 'known' sequences at either side of a newly proposed infection time, where a 'known' sequence can either be an observed or imputed sequence. With a current infection time E_j for an individual j, the key idea is to propose a new sequence at a proposed new infection time $E_j^{'}$ which has plausible proximity to a *nearest* **past** *sequence* $G_{\mathbf{p}}$ and a *nearest* **future** *sequence* $G_{\mathbf{f}}$ relative to $E_j^{'}$. We choose $G_{\mathbf{p}}$ and $G_{\mathbf{f}}$ by taking account of the sequences both from individual j and the source of infection ψ_j. Let $t_{\mathbf{p}}$ and $t_{\mathbf{f}}$ be the sequencing times for $G_{\mathbf{p}}$ and $G_{\mathbf{f}}$ respectively. Given the nucleotide base $G_{\mathbf{p}}^k$ and $G_{\mathbf{f}}^k$ at the kth position and $\Delta t_{\mathbf{p}} = |t_{\mathbf{f}} - t_{\mathbf{p}}|$, by assuming a linear relationship between the probability of change and the time duration, we have a newly proposed base at the kth position for the sequence at $E_j^{'}$ on individual j

$$g = \begin{cases} G_{\mathbf{f}}^k, & \text{with probability } P_{\mathbf{f}} = \frac{\left|E_j^{'} - t_{\mathbf{p}}\right|}{\Delta t_{\mathbf{p}}}, \\ G_{\mathbf{p}}^k, & \text{with probability } 1 - P_{\mathbf{f}}. \end{cases} \tag{1}$$

The source of infection is also updated in a similar fashion [3].

Other model parameters such as infection rates α and β are inferred using standard MCMC algorithm. Given inferred model quantities (including m the number of observed mutation), latent residual sample $\widetilde{r'}$ can be obtained by reversing the procedure described in Equation 5 in the main text. Non-informative uniform priors with wide intervals are used for all model parameters. S1 Table lists the prior distributions used for fitting the model to the FMD data.

Refining S-D-S Model $\boldsymbol{M}_0$

Pseudo-likelihood Function We propose a likelihood function for the within-host-diversity 'emulator' $\boldsymbol{M}_p$ for calibrating the single-dominant-strain model $\boldsymbol{M}_0$. The likelihood function accounts for the complete genetic data by augmenting the genetic data with the unknown $T^*(G_A, G_B)$ (see Equation 9 in main text) for each successive pair of transmitted strains. We describe briefly how this likelihood is constructed.

- Consider the a chain of infection events − individual k is infected at t_k, and it subsequently infects individual i at t_i and then j at t_j $(t_j > t_i > t_k)$.
- Let the dominant sequence at t_k on k be G_0^k, the sequence at t_i on i be G_0^i and the sequence at t_j on j be G_0^j. Noted that as we assume that the dominant strain on an infector will also be transmitted to a new infected individual, we have $G_1^k = G_0^i$ and $G_2^k = G_0^j$ − accordingly the sequence data on k may be denoted as $\boldsymbol{G}^k = (G_0^k, G_1^k = G_0^i, G_2^k = G_0^j)$.
- Each infection (i.e., k, i and j) is associated with a $T_l^* \sim \text{Beta}(\gamma, \eta)$, where $l = k, i$, or j. T_l^* will be used to determine the effective genetic time between the latest sequence on the infector (by the time of the infection) and the sequence on the infected individual (at the time of the

infection). For example, for the infected individual j, the corresponding effective genetic time between G_0^j and G_0^i

$$T(G_1^k = G_0^i, G_2^k = G_0^j) = 2 \times T_j^* \times (t_i - t_k) + (t_j - t_i) \tag{2}$$

Then, for individual k, we can construct a pseudo-likelihood function as follows.

$$\begin{aligned} L(\gamma, \eta, \lambda; \boldsymbol{G}) \propto & \prod_{l=i,i,k} P_B(T_j^*|\gamma, \eta) \times \\ & \prod_{m=0}^{1} P_T(G_m^k, G_{m+1}^k|\lambda, T(G_m^k, G_{m+1}^k)), \end{aligned} \tag{3}$$

where P_B is the density of Beta distribution and λ is the mutation rate and

$$P_T(G_m^k, G_{m+1}^k|\lambda, T(G_m^k, G_{m+1}^k)) = 1 - e^{-\lambda T(G_m^k, G_{m+1}^k)}.$$

Noted that Equation 3 only described a likelihood function for the evolutionary process − a complete likelihood function requires components that account for the epidemiological process which can be found in [3]. Equation 3 is in effect a pseudo-likelihood as it assumes that the relationship of G_{m+1}^k to G_m^k is independent of the latter's relationship to any previous strains. Compared to $\boldsymbol{M}_0$, this approach allows the effective time between strains to be an additional imputed quantity by inferring γ and η, and therefore allows departure from the more restricted s-d-s assumption made in $\boldsymbol{M}_0$.

Model Inference Model inference is performed by adapting the MCMC algorithm used to fit the s-d-s model in [3] (see an overview above in *Bayesian Data-augmentation and Model Inference of S-D-S Model* $\boldsymbol{M}_0$), by further augmenting the T^* (and the effective genetic time) and replacing the part of the likelihood contributed by the evolutionary process with the pseudo-likelihood function specified in Equation 3 above.

Specifically, for a particular infected host i, a new vector $\boldsymbol{v} = (\psi_k, t_i, G_0^i)$ are first jointly proposed, where ψ_i the source of infection, t_i the time of infection and G_0^i the dominant sequence at time of infection, conditional on current values of other model parameters including the T^*. The main idea for such joint proposal follows [3] largely − i.e., after proposing a new source and new time of infection, propose a new and statistically consistent sequence somewhere between two 'known' sequences (namely the *nearest past* sequence and the *nearest future* sequence) at either side of a newly proposed infection time, where a 'known' sequence can either be an observed or imputed sequence. Noted that effective genetic time, associated with the proposed G_0^i and the latest sequence on the proposed infector by the proposed time t_i, is also simultaneously updated (See Equation 2). The effective times between the (to-be-proposed) G_0^i and the two 'known' sequences at either side G_0^i are to used to determine the probability that a nucleotide base on G_0^i takes the value on the nearest past/future sequence at the same position. Acceptance probability of the proposed $\boldsymbol{v}$ and the associated effective genetic time is determined similarly following [3].

The algorithm is then applied sequentially to all infected hosts in each MCMC iteration, so that the whole set of effective genetic times, sources of infection, infection times and transmitted sequences may be updated. Furthermore, at each iteration, the collection of T^* for each infection is also updated using a Metropolis-Hastings sampler where new T^* is proposed using the Beta distribution parameterized by the current values of γ and η. The parameters γ and η are proposed using random-walk centered at current values. We use the same set of prior distributions for the shared parameters found in the s-d-s model (S1 Table), and additionally, we have non-informative prior $Exponential(rate = 0.01)$ for parameters γ and η.
