# Supplementary figures and images for: Model diagnostics and refinement for phylodynamic models
Source: PLoS Comput Biol. 2019 Apr 5;15(4):e1006955. doi: 10.1371/journal.pcbi.1006955 (PMC6469796; doi:10.1371/journal.pcbi.1006955)

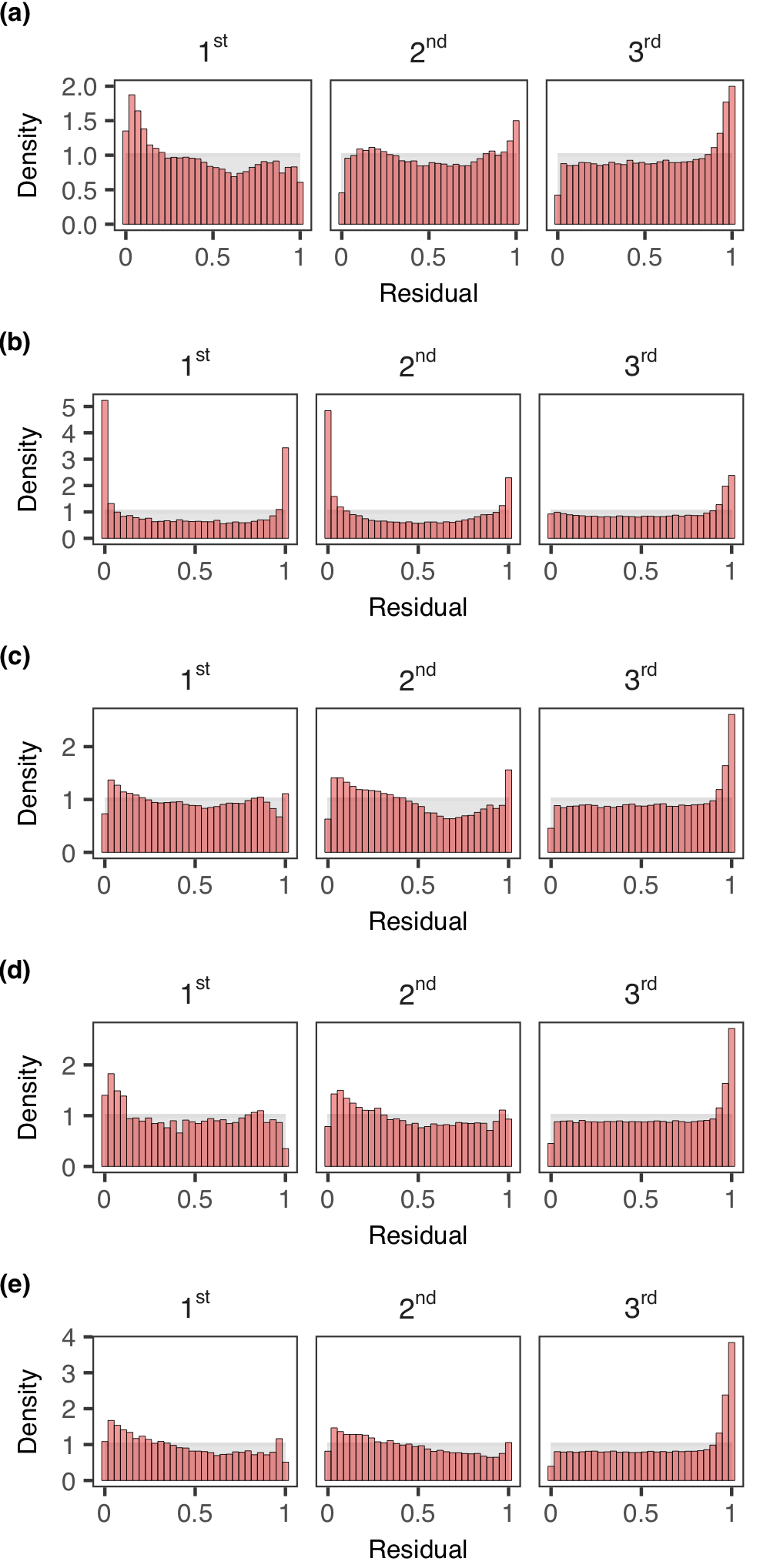

Supplement: S1 Fig — (a)-(e) correspond to simulation set 1-5. Each set of the imputed residuals r′˜ are first ordered according to the mark ζ(k). The ordered residuals are then subdivided into three equal-size samples − 1st, 2nd and 3rd one-third. Residuals associated with smaller ζ(k) (1st ans 2nd) exhibit a multiplicity of patterns, as opposed to the 3rd one-third where deviation is consistently observed at the right-tail of the unit interval (0, 1) (see also main text). (TIFF) [file pcbi.1006955.s002.tiff]

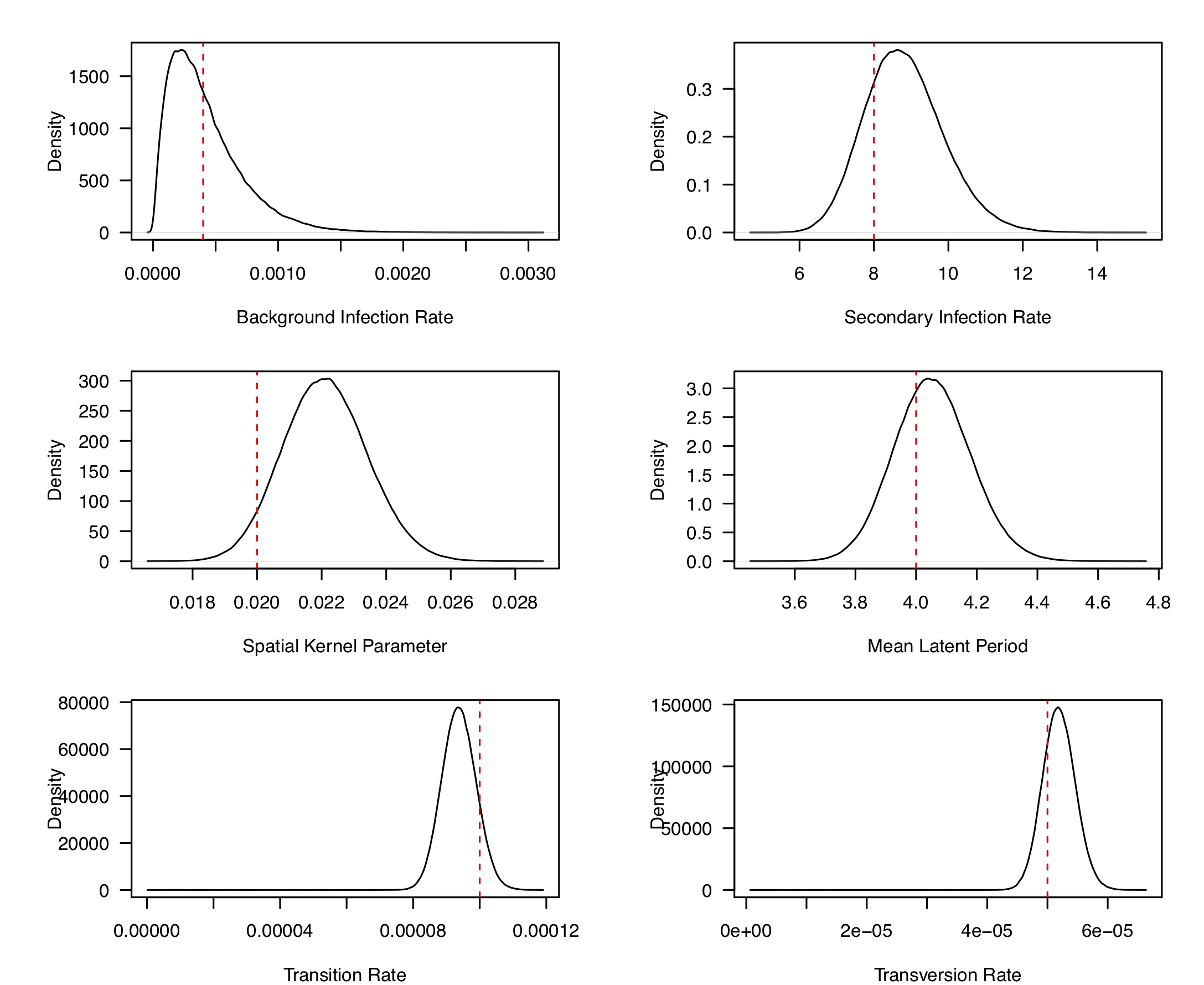

Supplement: S2 Fig — (TIFF) [file pcbi.1006955.s003.tiff]

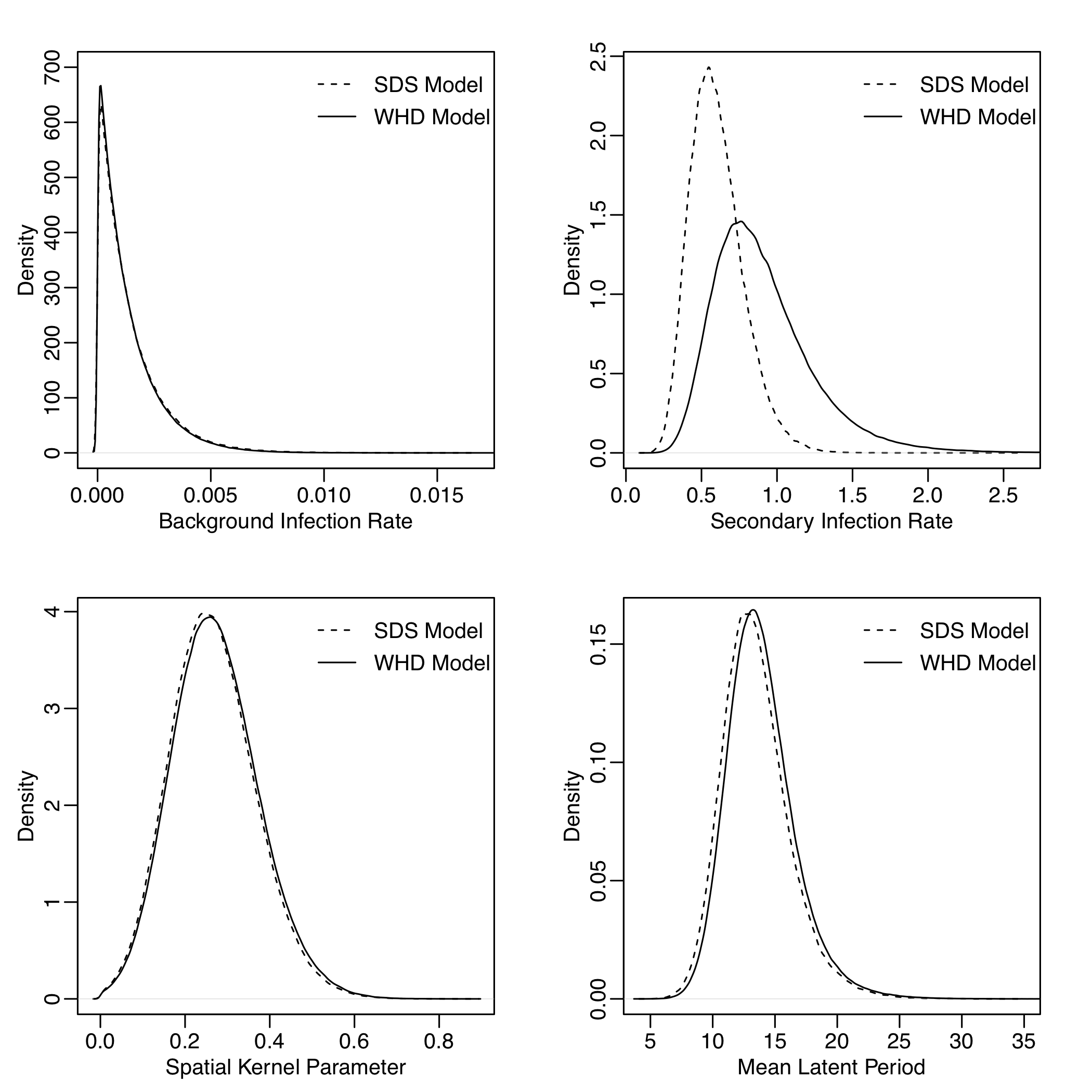

Supplement: S3 Fig — (TIFF) [file pcbi.1006955.s004.tiff]

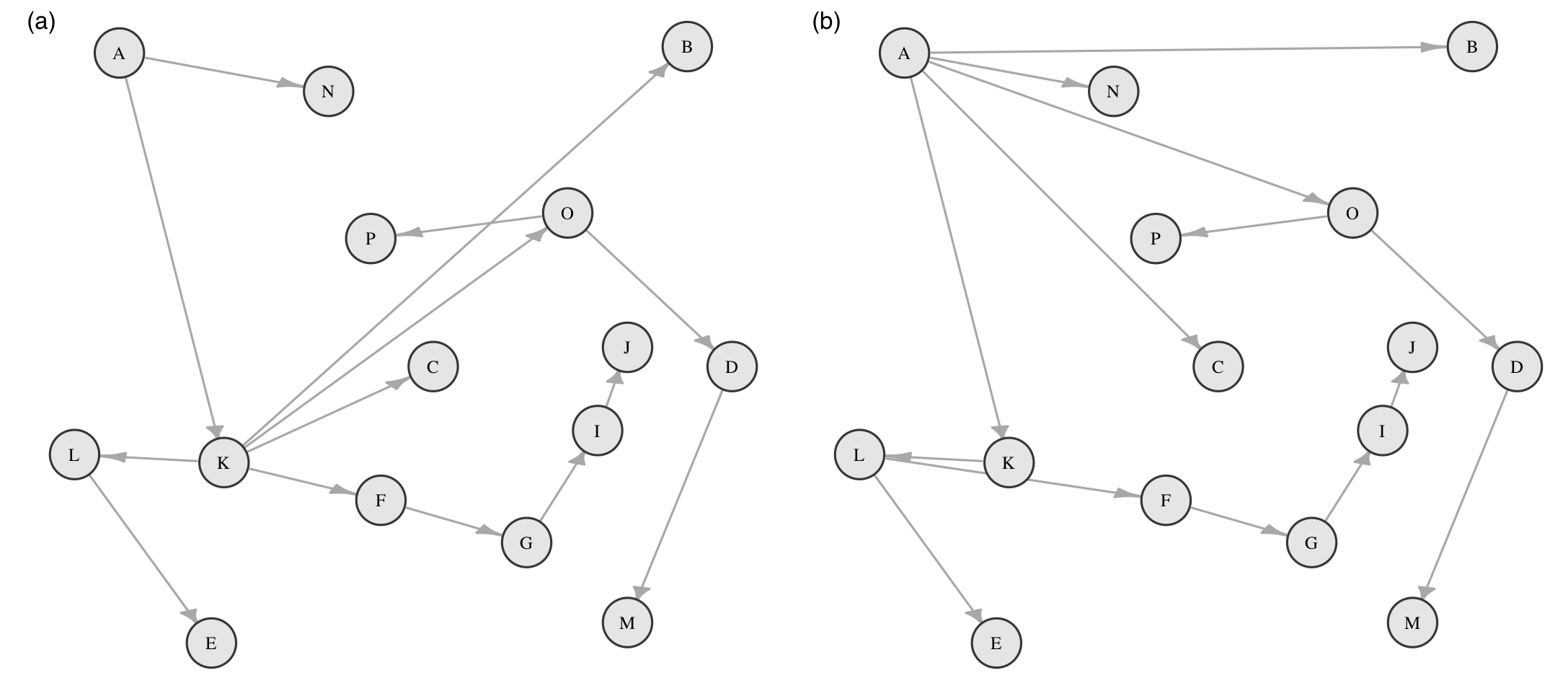

Supplement: S4 Fig — (a) Obtained from fitting the s-d-s model; (b) Obtained from fitting the pseudo-likelihood model. (TIFF) [file pcbi.1006955.s005.tiff]
