# Supplementary material for: Model diagnostics and refinement for phylodynamic models
Source: PLoS Comput Biol. 2019 Apr 5;15(4):e1006955. doi: 10.1371/journal.pcbi.1006955 (PMC6469796; doi:10.1371/journal.pcbi.1006955)
Supplement: S1 Table — (PDF) [file pcbi.1006955.s006.pdf]

Supporting Information

S1 Table

Parameter	Prior
κ, spatial kernel parameter	U(0,20)
β, secondary transmission rate	U(0,50)
α, background infection rate	U(0,0.5)
a, shape parameter of the latent period	U(0,50)
b, scale parameter of the latent period	U(0,30)
μ, mean of the infectious period	U(0,100)
μ_1, nucleotide transition rate	U(0,1)
μ_2, nucleotide transversion rate	U(0,1)
